# 

**Published:** 1849-09

**Authors:** 


### WESTERN JOURNAL

OF

### MEDICINE AND SURGERY:

EDITED BY

LUNSFORD P. YANDELL, M. D.,

PROFESSOR OF PHYSIOLOGY AND PATHOLOGICAL ANATOMY IN THE UNIVERSITY OF LOUISVILLE,

AND

THEODORE S. BELL, M. D.

---

CXVII.—SEPTEMBER, 1849.

---

THIRD SERIES.

VOL. IV.....NO. III.

---

LOUISVILLE, KY:

PUBLISHED MONTHLY BY PRENTICE AND WEISSINGER,

PROPRIETORS AND PRINTERS.

1849.

